# Concordance Between Clinical and Pathological Response Assessment After Neo-Adjuvant Chemotherapy in Patients With Invasive Lobular Carcinoma

**DOI:** 10.7759/cureus.14341

**Published:** 2021-04-07

**Authors:** Aisha Shaikh, Muhammad Usman Tariq, Shaista Masood Khan, Romana Idress, Lubna M Vohra, Saira Fatima Shaikh, Hira Waheed

**Affiliations:** 1 Surgery, Aga Khan University Hospital, Karachi, PAK; 2 Surgery, Shaheed Mohtarma Benazir Bhutto Medical University, Larkana, PAK; 3 Histopathology, Pathology and Laboratory Medicine, Aga Khan University Hospital, Karachi, PAK; 4 Breast Surgery, Aga Khan University Hospital, Karachi, PAK; 5 Histopathology, Aga Khan University Hospital, Karachi, PAK; 6 Radiology, Aga Khan University Hospital, Karachi, PAK

**Keywords:** neo-adjuvant, chemotherapy, response, lobular carcinoma, concordance

## Abstract

Background

Neo-adjuvant chemotherapy (NAC) is frequently administered in breast carcinoma patients. The clinical response to NAC guides further treatment. The pathological response is not only an independent prognostic factor, but it also guides further treatment and prognosis.

Objectives

The aim of our study was to find the degree of concordance between clinical and pathological response assessments after NAC in Invasive lobular Carcinoma (ILC) cases by using World Health Organization (WHO) criteria and different pathological systems, respectively. We also tried to identify any useful parameter of clinical assessment that could better correlate with pathologic assessment and provide a better estimation of residual tumor.

Methods

This retrospective study was conducted on 26 ILC tumors diagnosed in 24 patients who were treated with NAC followed by surgical resection between January 2009 and December 2020. Medical records and microscopy glass slides were reviewed for clinical and pathological response assessments, respectively.

Results

The pre-treatment tumor area ranged from 1.8-255 cm^2^ and the mean±SD was 52.2±66.8 cm^2^. After NAC, complete clinical response was observed in four (15.3%) cases. The clinically assessed mean tumor area significantly reduced from 52.2±66.8 cm^2^ to 17.2±22.6 cm^2^ (*p-*value<0.001). The pathologically assessed mean tumor area (27.4±24.1 cm^2^) didn’t differ significantly from the clinically assessed mean tumor area (17.2±22.6 cm^2^) (*p-*value=0.114). Pathologically, the majority of the cases showed partial response, and a complete pathological response was achieved in only two (7.7%) cases. The concordance rates between clinical assessment by the WHO method and pathological assessment of the breast using the Sataloff method, Miller-Payne (MP) system, Residual Cancer Burden system, and Chevallier method were 26.7%, 15.8%, 9%, and 3.5%, respectively, with insignificant *p*-values. Percentage reduction in clinical size and percentage reduction in tumor cellularity differed significantly (*p*-value=0.038).

Conclusion

Clinical response assessment provides a less accurate estimation of residual disease, as it shows poor concordance with pathological assessment using different assessment systems/methods.

## Introduction

Invasive lobular carcinoma (ILC) is the second most common type of breast carcinoma with distinct clinical, biological, and prognostic features [1-2]. ILC is usually clinically deceptive and radiologically less distinctive due to the diffusely infiltrative growth pattern. Histological evaluation of the excision specimen reveals tumor sizes larger than clinical and radiological assessments [1-5]. Neo-adjuvant chemotherapy (NAC) is used frequently in locally advanced breast cancers (LABC) to improve the operability of the tumor [1-2,4,6]. Response to chemotherapy predicts prognosis and guides further treatment [4-8]. Since the clinical assessment of the response may be compromised by chemotherapy-related fibrosis; radiological evaluation aids in the assessment of residual tumor [1-3,6]. Around 70% of breast carcinoma shows clinical and radiological response but only 3%-40% of cases achieve complete pathological response [4-7,9]. Histological evaluation is considered an independent prognostic factor and the gold standard method for response assessment [4-7,8,10-11].

Different clinical and pathological criteria are used to grade clinical and pathological responses after NAC [5,7-9,12]. Clinical response assessment methods include WHO and RECIST (Response Evaluation Criteria in Solid Tumors) criteria. WHO criteria have been widely practiced for standardized tumor response evaluation [5,7,9-10]. It measures two maximum perpendicular dimensions of the tumor mass before and after NAC. The extent of tumor shrinkage is determined by the percentage change in the product of these dimensions [5,7,10].

Methods used to evaluate pathological response include the Miller-Payne (MP) system, Residual Cancer Burden system, National Surgical Adjuvant Breast and Bowel (NSABPB-18), Chevallier method, Sataloff method, etc. [7-9,13]. These systems have demonstrated a correlation between pathologic response and survival [8,11,14].

 ILC is less responsive to NAC, which results in higher rates of positive surgical margins and re-resections or completion mastectomies as compared to invasive ductal carcinoma (IDC) [1-3,6,11,15-17]. Researchers are attempting to identify methods and tools of response assessment that can accurately determine residual disease burden pre-operatively in order to select effective treatment. There is a paucity of research related to NAC response assessment in ILC.

This study was conducted with the aim to assess the patterns of clinical and pathological response to NAC in ILC cases by using WHO criteria and the Miller-Payne (MP) system, Residual Cancer Burden (RCB) system, Chevallier method, Sataloff method, respectively. We attempted to evaluate the concordance and agreement between these assessment systems. We also tried to identify any useful parameter of clinical assessment, which could better correlate with pathologic assessment and provide a better estimation of residual disease.

## Materials and methods

This retrospective cross-sectional was approved by the institution’s Ethical Review Committee. A total of 120 patients of ILC were diagnosed between January 2009 and December 2020. Only 24 patients with a non-metastatic disease received NAC for downstaging/downsizing of the primary tumor followed by surgery and were identified as the sample for the study. Patients with stage IV disease at the time of diagnosis and others who received chemotherapy in adjuvant settings were excluded from the study. Two of these patients had bilateral tumors. Data regarding patient’s age, gender, family history, tumor location, multicentricity/multifocality, clinically assessed pre & post-treatment tumor area (product of two larger dimensions), and pre and post-treatment clinical stage (cTN) were obtained from medical records. The percentage change in pre and post-treatment clinically assessed tumor area was graded according to WHO criteria [6].

Pre-treatment trucut biopsies were reviewed for tumor type, histologic grade, and tumor cellularity. Post-treatment specimens were reviewed for the histologic grade, residual tumor bed size, size of largest focus of invasive carcinoma, in-situ component, and lymph node metastasis. Cellularity of the residual tumor was compared with tumor cellularity in the pre-treatment trucut biopsy and the percentage difference (reduction) in cellularity was noted. Post-chemotherapy response in the breast was assessed according to the Miller-Payne system, Chevallier method, Sataloff method, and Residual Cancer Burden system. The response in lymph nodes was also categorized according to the Sataloff method [13]. The concordance rate between clinical and pathological assessment was determined by calculating kappa statics and expressed as a percentage. A p-value of <0.05 was considered significant.

## Results

Twenty-six tumors from 24 patients were included in the study. Patient demographics, pre-treatment tumor characteristics, and treatment information are summarized in Table 1.

**Table 1:** Summary of patient demographics, pre-treatment tumor characteristics, and treatment information (24 patients and 26 tumors) *AJCC: American Joint Committee on Cancer; ER+PR: estrogen and progesterone receptor

Characteristics	Expression
Patient’s age (years):	
- Range	32-76
- Mean±SD	50±11
- Median	49
Tumor location:	
- Upper outer quadrant	21(80.7%)
- Lower inner quadrant	2(7.7%)
- Retro-areolar	2(7.7%)
- Upper inner quadrant	1(3.8%)
Number of lesions:	
- Single	17(65.4%)
- Multiple	9(34.6%)
Pre-treatment tumor grade:	
- Grade I	1(3.8%)
- Grade II	18(69.2%)
- Grade III	7(26.9%)
Tumor biology:	
- ER+PR positive, Her2Neu negative	21(81%)
- Triple positive	3(11%)
- Triple negative	2(7.7%)
Clinically-assessed tumor area (cm^2^):	
- Range	1.8-255
- Mean±SD	52.2±66.8
- Median	25.4
AJCC* clinical T stage:	
- T1	2(7.7%)
- T2	1(3.8%)
- T3	11(42.3%)
- T4	12(46.2%)
Clinical N stage:	
- N0	1(3.8%)
- N1	17(65.4%)
- N2	7(26.9%)
- N3	1(3.8%)
Chemotherapy regimen:	
- Anthracycline and taxane based	22 (85%)
- Anthracycline only	1 (4%)
- Others	3 (11%)

All of the patients were female. Nine (34.6%) cases had a family history of breast carcinoma. Tumors with HER-2 positive status also received targeted anti-HER-2 therapy in addition to chemotherapy. Patients with positive hormone receptor status received oral tamoxifen therapy for five to seven years. Oncotype DX testing was not performed preoperatively in any of these patients, as this facility was not available in the country when these patients were treated. All of the patients underwent mastectomy along with axillary lymph node sampling/clearance. The major reasons were ineligibility for breast-conserving surgery (BCS) at the outset due to the multi-centric nature of tumors and refusal of patients for breast conservation. The post-treatment tumor characteristics are summarized in Table 2.

**Table 2:** Summary of post-treatment tumor characteristics and treatment response (n=26) *AJCC: American Joint Committee on Cancer

Characteristics	Expression
Clinical response assessment (WHO criteria):	
- Complete response (No residual tumor)	4(15.4%)
- Partial response (≥50% decrease in size)	13(50%)
- Stable disease (<50% decrease in size)	9(34.6%)
- Progressive disease (>25% increase in size)	0
Clinically-assessed post-treatment tumor area (cm^2^):	
- Range	0-85.5
- Mean±SD	17.2±22.6
- Median	10.5
Difference in pre & post-treatment clinically-assessed tumor areas (cm^2^):	
- Range	2.5-246
- Mean±SD	34.9±59
- Median	12.2
Pathologically-assessed residual tumor area (cm^2^):	
- Range	1-82.5
- Mean±SD	27.4±24.1
- Median	15.3
Size of largest focus of invasive carcinoma (cm):	
- Range	0.4-11
- Mean±SD	3.5±3
- Median	2.5
Percentage loss of tumor cellularity (pathological):	
- Range	15-100%
- Median	47.5%
AJCC* post-treatment pathologic T stage:	
- T0	2 (7.7%)
- T1	9 (34.6%)
- T2	8 (30.8%)
- T3	7 (27%)
- T4	0
Status of lymph nodes:	
- Positive	19 (73%)
- Negative	7 (27%)
Extra nodal extension:	
- Present	12 (63.1%)
- Absent	7 (36.9%)
Treatment effect in lymph nodes:	
- Present	11 (42.3%)
- Partial	10
- Complete	1
- Absent	15 (57.7%)
AJCC* post-treatment pathologic N stage:	
- N0	7 (27%)
- N1	4 (15.4%)
- N2	12 (46.1%)
- N3	3 (11.5%)

According to WHO criteria, clinical response was appreciated in 17 (65.4%) cases. Residual tumor was identified in 24 cases and all of these cases showed a loss in tumor cellularity and at least partial response (Table 2). One of the cases exhibiting pathologic complete response was a clinical-stage T3 tumor with grade III morphology on trucut biopsy. The other case was a clinical-stage T2 tumor with grade II morphology on trucut biopsy. Both cases were hormone receptor-positive and Her2neu negative. Residual tumor categorization according to different pathologic response assessments is summarized in Table 3.

**Table 3:** Pathologic response assessment using different systems (n=26)

Assessment systems and their categories	Frequency (%)
Miller-Payne system:	
- Grade 1 (No response)	0
- Grade 2 (Partial response; up to 30% loss)	6(23%)
- Grade 3 (Partial response; 30-90% loss)	15(57.6%)
- Grade 4 (Almost complete response; >90% loss)	3(11.5%)
- Grade 5 (Complete response)	2(7.7%)
Residual Cancer Burden (RCB) System:	
- RCB-0 (No carcinoma in breast or lymph node)	02 (7.7%)
- RCB-I (Partial response)	0
- RCB-II (Partial response)	6 (23%)
- RCB-III (Chemoresistant)	18 (69.2%)
- Range of score	0 - 4.632
- Median score	3.707
Chevallier Method:	
- Class 1 (Pathologic Complete Response)	02 (7.7%)
- Class 2 (Pathologic Complete Response)	0
- Class 3 (Pathologic Partial Response)	24 (92.3%)
- Class 4 (Pathologic No Response)	0
Sataloff Method for Tumor:	
- T-A (total or near-total therapeutic effect)	05 (19.2%)
- T-B (≥ 50% therapeutic effect)	08 (30.8%)
- T-C (< 50% therapeutic effect)	13 (50%)
- T-D (no therapeutic effect)	0
Sataloff Method for Lymph Nodes:	
- N-A (Evidence of therapeutic effect, no metastatic disease)	1 (3.8%)
- N-B (No nodal metastasis or therapeutic effect)	6 (23%)
- N-C (Evidence of therapeutic effect but nodal metastasis present)	10 (38.5%)
- N-D (Viable metastatic disease, no therapeutic effect)	09 (34.6%)

The tumor’s histologic grade was upgraded in four (15.4%) and downgraded in four (15.4%) cases. Overall, the clinical response rate was 65.38% and a pathologic (partial or complete) response rate of 100%. Poor concordance was observed between clinical and pathological response assessments using different systems (Table 4).

**Table 4:** Concordance between clinical response assessment according to WHO criteria and pathological response assessment according to different systems (n=26) *Number of concordant cases is highlighted; **S.E: standard error; ***C.I: confidence interval WHO: World Health Organization

Pathological response assessment system	Clinical response assessment (WHO criteria)			Kappa values, Confidence interval (C.I) & p-value
	Complete response (n=4)	Partial response (n=13)	Stable disease/ No response (n=9)	
Miller-Payne system:				
Grade 1	0	0	0	Kappa coefficient = 0.158
Grade2	0	5(19.2%)	1(3.8%)	S.E** of kappa = 0.109
Grade 3	3(11.5%)	6(23.1%)	6(23.1%)	95% C.I***: -0.056 - 0.372
Grade 4	0	1(3.8%)	2(7.7%)	p-value: 0.685
Grade 5	1(3.8%)	1(3.8%)	0	
Residual Cancer Burden System (RCB):				
RCB-0	0	2(7.7%)	0	Kappa coefficient = 0.09
RCB-I	0	0	0	S.E of kappa = 0.066
RCB-II	2(7.7%)	2(7.7%)	2(7.7%)	95% C.I: -0.22 - 0.04
RCB-III	2(7.7%)	9(34.6%)	7(26.9%)	p-value: 0.845
Chevallier Method:				
Class 1	0	2(7.7%)	0	Kappa coefficient = 0.035
Class 2	0	0	0	S.E of kappa = 0.022
Class 3	4(15.4%)	11(42.3%)	9(34.6%)	95% C.I: -0.021 - 0.065
Class 4	0	0	0	p-value: 0.865
Sataloff Method for Tumor:				
T-A	2(7.7%)	2(7.7%)	1(3.8%)	Kappa coefficient = 0.267
T-B	1(3.8%)	5(19.2%)	2(7.7%)	S.E of kappa = 0.148
T-C	1(3.8%)	6(23.1%)	6(23.1%)	95% C.I: -0.067 - 0.513
T-D	0	0	0	p-value:0.455
Sataloff Method for Lymph Nodes:	Assessment of lymph nodes (Not according to WHO criteria)			
	Disappeared	Decreased	No change	
N-A	0	1(3.8%)	0	Kappa coefficient = 0.067
N-B	4(15.4%)	1(3.8%)	1(3.8%)	S.E of kappa = 0.073
N-C	3(3.8%)	5(19.2%)	2(7.7%)	95% C.I: 0.07 - 0.093
N-D	2(7.7%)	5(19.2%)	2(7.7%)	p-value: 0.761

The Sataloff system for breast tumors showed a maximum concordance rate of 26.7% with WHO criteria, followed by the MP system (15.8%), RCB system (9%), and Chevallier method (3.5%). The Sataloff system for lymph nodes showed a concordance rate of 6.7%. None of these concordance rates was statistically significant. Despite poor concordance, clinically assessed and pathologically assessed mean tumor areas (17.2±22.6cm2 and 27.4±24.1cm2) were not significantly different when an independent-samples t-test was applied (p-value=0.114). However, the mean percentage change in clinical size (63.5±34.2%) was significantly higher from the pathologically assessed percentage reduction in tumor cellularity (52.9±27.3%) (p-value=0.038).

The clinical assessment of the triple-negative subgroup (n=2) revealed complete response in one case and partial response in the other case. Pathological assessment of this subgroup also revealed complete response in one case and partial response in the other case. Similarly, clinical and pathological assessments of the triple-positive subgroup (n=3) revealed partial response in all three cases. In hormone receptor-positive, the Her2neu negative subgroup (n=21), clinical complete response was observed in three cases, partial response in 14 cases, and no response in four cases. Pathologically, complete response was observed in one case and partial response in the rest of the 20 cases. Thus, maximum concordance between clinical and pathological response assessments was observed in the triple-negative and triple-positive subgroups. While the ER PR positive and Her2neu negative subgroups clinically revealed complete response and no response in a total of seven cases, which was discordant with pathological response assessment in six cases.

The follow-up duration ranged from 49-115 months. All of the patients were alive except for a single patient who died of a stroke. Two of the patients were hospitalized due to lymphedema.

## Discussion

The pathologic complete response rates for ILC range from 2%-13% as compared to 12%-35.5% for IDC [1-2,15,17]. Positive estrogen expression, Her2neu negativity, and low histologic grade are possible causes of low response [1,3,14,17]. Many criteria for response assessment have been evaluated by various studies [5,7-9,13-14]. A study tested whether WHO and RECIST criteria are interchangeable and concluded that, with slight modification in the cut-off of “RECIST” criteria, two criteria may be used interchangeably [5]. Our study used WHO criteria only as most of the tumors were ill-defined or multi-centric and, therefore, RECIST criteria were not applicable [5,10].

Alqahtani S et al. reported complete clinical response in 16.1% of cases [1]. Alqahtani S et al. and Turin W et al. reported a complete pathological response in 4.9% and 13% cases, respectively [1-2]. Similarly, we also observed complete clinical and pathological responses in 15.4% and 7.7% cases, respectively. Loibl S et al. reported that grade III and hormone receptor-negative tumors respond better to NAC [11]. In our experience, one of the tumors with complete pathological response was grade III while the other was grade II. But both of these tumors were strong hormone receptor-positive.

Different studies have found that clinical and pathological responses don’t correlate and a variable degree of disparity exists between response rates [1-2,4,7]. Response assessment in ILC is difficult due to the discohesive nature of tumor cells whereby neoplastic cells spread into the surrounding tissue in small clusters and thin linear cords, which are visualized microscopically but are not appreciated clinically and radiologically because of their non-compact (less dense) nature. In addition, reduction of tumor size and tumor replacement by fibrosis is considered a complete tumor response clinically and radiologically, but this assessment is usually not concordant with the pathological assessment since these areas may contain tiny clusters of residual tumor, which is considered a pathological partial response [1-2,4-5,15].

Another cause of disparity is the cut-off values used by the WHO criteria and pathological systems for categorizing the response. For example, a tumor having up to 50% reduction is considered “stable” by WHO criteria (non-responder) [6]. However, it is considered a “partial response” according to all pathological assessment systems [13]. These systems are not concordant among themselves, as they use different cut-off values; some include ductal carcinoma in situ (DCIS) in the assessment and some also include lymph node assessment [13,17]. To the best of our knowledge, this is the first study to statistically evaluate the concordance between clinical and pathological response by applying kappa statistics. We also identified poor concordance between clinical and pathological assessment using different systems. In our study, concordance rates between clinical and pathological response assessments ranged from 3.5% to 26.7%. Maximum concordance was observed between WHO criteria and the Sataloff method. The concordance rate for lymph nodes was poor, similar to that for breast. As an alternate approach, the mean of clinically assessed percentage reduction in tumor size and pathologically assessed percentage reduction in tumor cellularity were compared and the clinically assessed reduction was significantly higher than the pathologically assessed reduction, further confirming the disparity. When means of the clinically assessed and pathologically assessed tumor areas were compared, the clinically assessed mean tumor area was less than that assessed pathologically. The difference was statistically insignificant. Therefore, this parameter can be an alternative to get a better correlation between the clinical and pathological assessments.

The rate of breast-conserving surgery (BCS) in ILC is usually lower (9.7%-24.4%) than in IDC patients [1-2,15,17]. It is generally related to a higher rate of positive margins in ILC after NAC [15-17]. Many studies have reported a higher mastectomy rate in ILC patients, even those having a pathological complete response [1-3,5,11,15].

None of the patients in our study underwent BCS. The main reasons were the ineligibility for conservation and the patients’ refusal owing to fear of relapse and acceptance for mastectomy as a safe approach. Margins were clear in all of our cases.

This study has inherent limitations such as retrospective nature, small sample size, the difference in chemotherapy regimens, shorter follow-up duration, and single-institution experience.

## Conclusions

Clinical and pathological responses lack significant concordance using the available assessment system. Clinical complete response rate and clinically assessed percentage reduction in size is higher than the pathological response. Clinically and pathologically assessed mean tumor areas may prove to be a parameter with better correlation. Studies with a larger sample size are needed to validate and suggest better methods of response assessment.
